# SU5416, a VEGF Receptor Inhibitor and Ligand of the AHR, Represents a New Alternative for Immunomodulation

**DOI:** 10.1371/journal.pone.0044547

**Published:** 2012-09-06

**Authors:** Joshua D. Mezrich, Linh P. Nguyen, Greg Kennedy, Manabu Nukaya, John H. Fechner, Xiaoji Zhang, Yongna Xing, Christopher A. Bradfield

**Affiliations:** 1 Department of Surgery, University of Wisconsin School of Medicine and Public Health, Madison, Wisconsin, United States of America; 2 Division of Gastroenterology and Hepatology, School of Medicine, Stanford University, Stanford, California, United States of America; 3 Department of Oncology, McArdle Laboratory for Cancer Research, University of Wisconsin School of Medicine and Public Health, Madison, Wisconsin, United States of America; University of São Paulo, Brazil

## Abstract

The experimental compound SU5416 went as far as Phase III clinical trials as an anticancer agent, putatively because of its activity as a VEGFR-2 inhibitor, but showed poor results. Here, we show that SU5416 is also an aryl hydrocarbon receptor (AHR) agonist with unique properties. Like TCDD, SU5416 favors induction of indoleamine 2,3 dioxygenase (IDO) in immunologically relevant populations such as dendritic cells in an AHR-dependent manner, leading to generation of regulatory T-cells *in vitro*. These characteristics lead us to suggest that SU5416 may be an ideal clinical agent for treatment of autoimmune diseases and prevention of transplant rejection, two areas where regulatory ligands of the AHR have shown promise. At the same time, AHR agonism might represent a poor characteristic for an anticancer drug, as regulatory T-cells can inhibit clearance of cancer cells, and activation of the AHR can lead to upregulation of xenobiotic metabolizing enzymes that might influence the half-lives of co-administered chemotherapeutic agents. Not only does SU5416 activate the human AHR with a potency approaching 2,3,7,8-tetrachlorodibenzo-p-dioxin, but it also activates polymorphic murine receptor isoforms (encoded by the *Ahr^d^* and *Ahr^b1^* alleles) with similar potency, a finding that has rarely been described and may have implications in identifying true endogenous ligands of this receptor.

## Introduction

Agonists of the aryl hydrocarbon receptor (AHR) have been of interest to the pharmaceutical industry for many years. This interest originally stemmed from the observation that the AHR is a ligand-activated transcription factor that regulates the adaptive metabolism of xenobiotics [1] and because receptor binding is a known step in the carcinogenic and toxic action of environmental pollutants like 2,3,7,8-tetrachlorodibenzo-p-dioxin (TCDD) [2]. Thus, agonism of the AHR has commonly been considered a signature for drugs that upregulate phase-I and phase-II metabolic systems and also for chemicals with pharmacological similarity to a known human carcinogen. As a result, AHR agonism has largely been considered a hazard signature for environmental chemicals and drugs in the pharmaceutical pipeline.

Recent insights related to the normal physiological role of the AHR are changing our view of receptor agonism to one where agonism might be considered to hold therapeutic value. A number of recent reports are identifying new biological processes that might be influenced by endogenous receptor ligands. For example, descriptions of mice harboring a null allele at the *Ahr* locus indicate that receptor signaling plays an important role in normal cardiovascular development and function [3], [4]. The therapeutic potential related to this biology is demonstrated by the observation that potent AHR agonists like TCDD can correct developmental aberrations in hepatic blood flow under conditions of AHR hypomorphism [5].

More recently, a role for the AHR in immunology has been emphasized by reports that activation of this receptor with ligands, such as TCDD, can lead to the generation of regulatory T-cells (Tregs) [6], while activation with other ligands, such as formylindolo[3,2-b]carbazole (FICZ) can lead to Th17 cell formation [7]. The potential clinical importance of this finding is supported by the observation that TCDD is able to ameliorate the symptoms of experimental autoimmune encephalomyelitis (EAE) in mice, whereas FICZ aggravates this syndrome. Additional studies have supported the idea that ligands can play a role in improving allograft acceptance after transplantation [8]. The importance of the AHR in immunology has also been extended by a series of papers demonstrating the central importance of this receptor in the presence and maintenance of intraepithelial lymphocytes and lymphoid tissue inducer cells in the gut, highlighting that the AHR and its ligands play a role in normal physiology of the immune system and response to the outside environment [9], [10], [11].

We have begun a search for agonists and antagonists of the AHR as part of an effort to develop a new class of receptor ligands with therapeutic potential for the treatment of vascular or immunological disease. Our initial strategy is to screen compounds that are pharmacologically well studied and that pose less environmental or health risks as compared to TCDD. Our approach to initially screen a library of compounds with known biological activity (KBA) was chosen for three reasons. First, well studied compounds hold greater probability of prior toxicological and pharmacological characterization and thus may move into clinical settings more quickly. Second, identification of AHR ligands in classes of pharmacologically active compounds already in the clinic could shed additional insights into their mode of action, as well as identify compounds with understandable off-target effects. Third, pharmacological information about novel AHR agonists could provide insight into the endogenous mechanism of action of this receptor or reveal the biological pathways in which the receptor participates during development.

As one result of this effort, we have discovered that [3-(3,5-dimethyl-1H-pyrrol-2-ylmethylene)-1,3-dihydro-indole-2-one] (SU5416), a known VEGFR-2 kinase inhibitor that progressed to Phase III clinical trials for metastatic colorectal cancer, is also a potent AHR agonist, active in a variety of mammalian systems. This new understanding of the dual signaling of SU5416 has implications for future clinical trials and may provide promise for the direction of future efforts aimed at diseases particularly well suited for such a pharmacologically unique compound.

The findings in this manuscript will identify two novel concepts that will help us understand the role of the AHR in normal physiology and be translatable clinically. First, we will discuss the possibility that the AHR can be considered as a target for immune modulation and treatment of diseases including autoimmunity and transplant rejection, and paradoxically, also potentially for cancer therapy depending on the ligand employed. Based on efforts at characterizing novel ligands of the AHR in relation to their interaction with the acquired immune system, we envision that ligands can either be “regulatory” or “effector”, depending on the inflammatory milieu and dosing strategies of the ligands. In the future this may form the basis for an entirely new class of drugs targeting the AHR for immunomodulation.

A second novel concept in this manuscript is the ability of SU5416 to activate the AHR^b^ and AHR^d^ polymorphisms with similar efficacy. These two isoforms are present in different strains of mice, and have been well characterized for many ligands, particularly TCDD. For the majority of ligands studied, the AHR^d^ isoform displays less than one-tenth the response of AHR^b^ after binding. It has been proposed that a true endogenous ligand of the AHR would activate the two polymorphisms similarly, given the importance of the AHR in normal physiologic development, and that mice with either genotype do not display the abnormal phenotypes seen in AHR^−/−^ and hypomorphic mice [12]. While we initially utilized the AHR^d^ polymorphism to narrow our search for potent ligands of the AHR, we inadvertently found that SU5416 activates these two isoforms with similar potency. This not only confirms the importance of this property of the drug in humans, who harbor the AHR^d^ polymorphism, but also will allow the structure of SU5416 to serve as a model in our search for clinically relevant endogenous ligands of the AHR.

## Results

### Primary Screen for Agonists of the Human AHR

**Figure 1:**
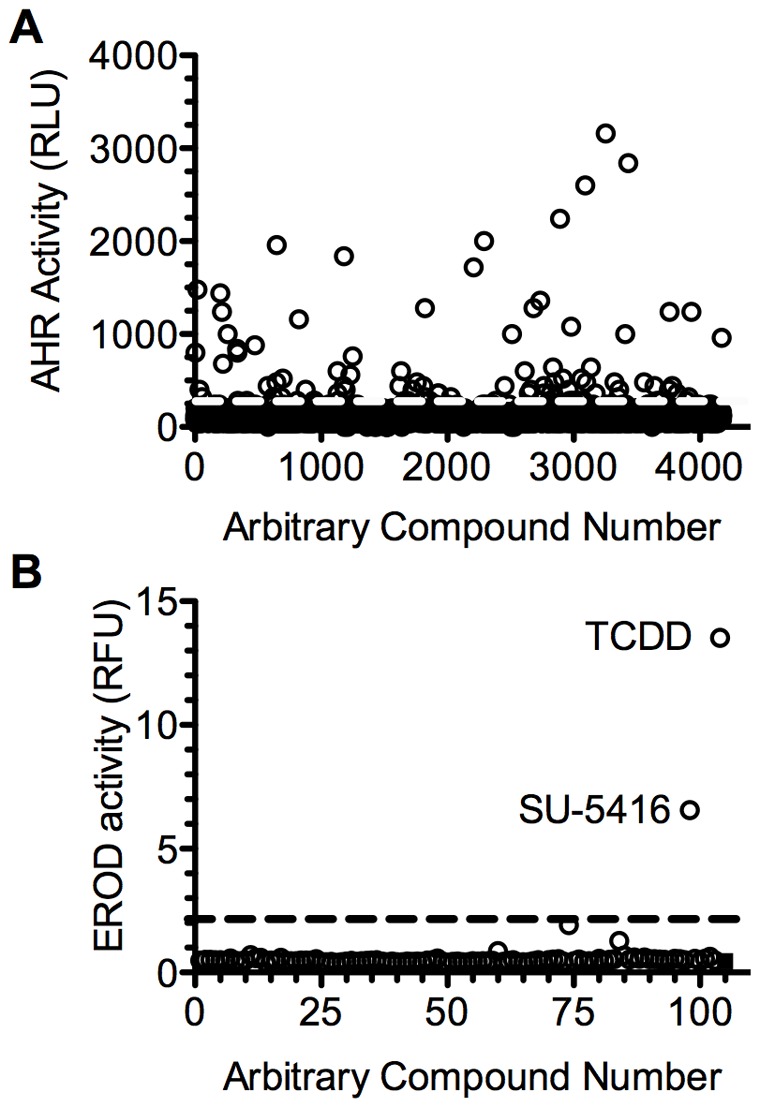
Screen of small molecule library for AHR agonists. **A.** A collection of 4160 compounds was screened for the induction of the DRE-driven luciferase in the human hepatoma 101L cell line. In 384-well plates, 100 µL media containing 70% confluent 101L cells was incubated with 10 µM of each test compound (1% v/v DMSO) for 24 hours. Dotted line indicates 3-fold induction. **B.** Screen for agonists of the AHR^d^. The AHRd-15 cell line was treated with 1 µM of the 98 compounds identified from the primary screen, 2 nM TCDD or DMSO and EROD activity was determined. Dashed line indicates 5-fold induction.

To identify novel agonists of the AHR, a library of 4,160 small molecules, “The KBA library”, was screened at 10 µM per compound, by the Small Molecular Screening Facility of The Carbone Cancer Center of the University of Wisconsin School of Medicine and Public Health. This library represents the sum of three commercially available well characterized chemical libraries with a high frequency of approved drugs and prototype signaling molecules. This includes 2,000 diverse FDA approved drugs and natural products (Microsource Discovery Systems, Inc; Gaylordsville, CT); the 1280 compound LOPAC^1280^ library of diverse characterized compounds (Sigma; St Louis, MO); and 880 characterized compounds (Prestwick Chemicals; Illkirch, FR). In this first stage of the screen, AHR agonism was determined by monitoring the activation of the human receptor using the human 101L-hepatoma cell line that has a stably integrated “dioxin-responsive element (DRE) driven luciferase reporter [13]. At the tested concentration of 10 µM, approximately 100 compounds induced at least a three-fold increase in luciferase activity (figure 1A).

### Secondary Screen for Agonists of the Murine AHR^d^ Low Affinity Receptor

The 100 “hit compounds” from the primary screen were subsequently screened for their capacity to activate the low-affinity murine AHR^d^ receptor isoform using the activity of the endogenous *Cyp1a1* gene as a readout. To this end, we established a hepatoma cell line that expresses the AHR^d^ receptor isoform derived from the DBA/2J mouse [14]. An AHR^d^-expressing cell line was generated by stably transfecting the AHR^d^ cDNA into the rat hepatoma AHR-deficient cell line, BP8 [15]. After stable selection with G418, a subclone (AHR^d^-15) was analyzed for receptor expression and function. First, a western blot using an anti-AHR antibody, revealed that the AHR^d^-15 cells produced an immunoreactive protein band that co-migrated with a receptor species isolated from the hepatic cytosol of DBA/2J mice (approximate size 104 kDa). This band was distinct from the AHR^b^ isoform found in C57BL/6J cytosol, which migrated at 97 kDa (figure S1A). To confirm that the AHR^d^-15 clone expressed a functional low affinity AHR^d^ isoform, we examined the receptor-mediated response to the prototype agonists, TCDD and β-naphthoflavone (BNF). Increasing concentrations of TCDD induced CYP1A1-mediated EROD activity in these cells with an EC_50_ in the 30 nM range [16]. In contrast, the much weaker agonist, BNF, known not to induce an AHR-mediated response in the AHR^d^ receptor isoform expressed in the hepatocytes of DBA/2J mice [17], was shown to be inactive at doses as high as 10 µM in the AHR^d^-15 cells (figure S1B).

To test the ability of the 100 AHR inducers to activate the AHR^d^-15 cells, they were treated with each of the compounds at the dose of 1 µM, for 36 hours in 96-well plates. Only the compound SU5416, and the positive control, TCDD, induced AHR^d^-mediated EROD activity greater than 5-fold (figure 1B). Therefore, SU5416 was considered for further analysis.

### Induction of DRE-mediated Transcription by SU5416 is AHR and ARNT Dependent

**Figure 2:**
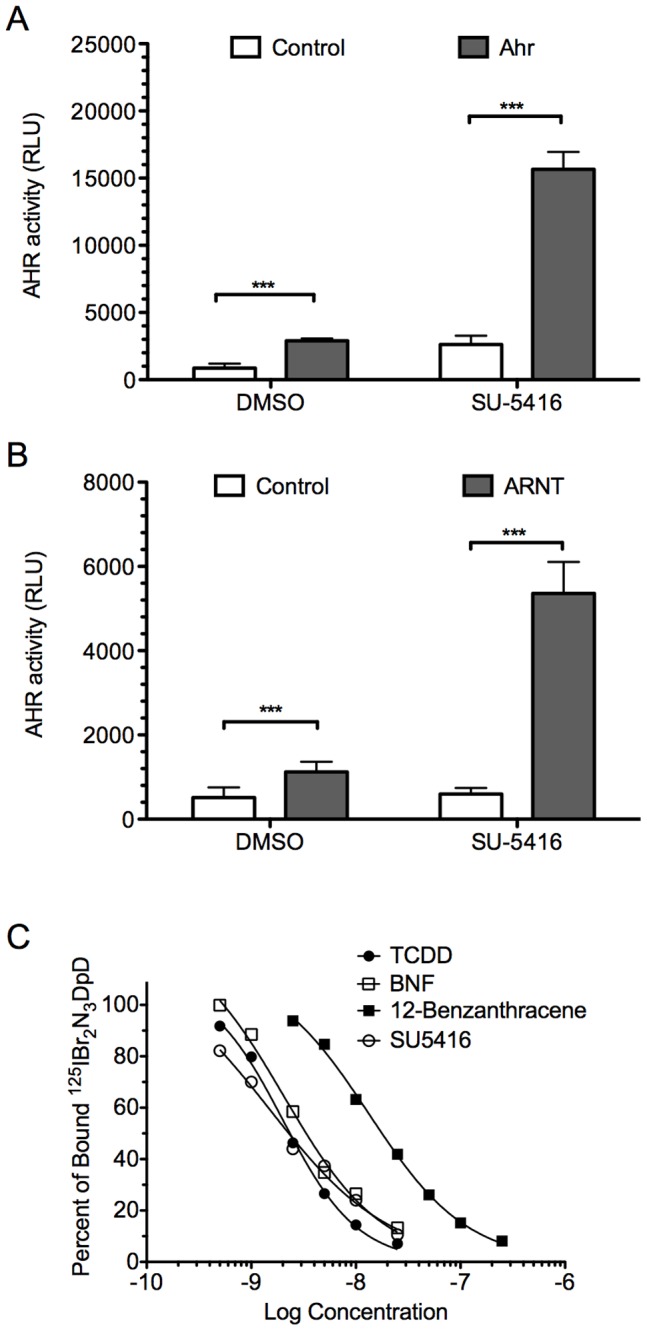
Induction of DRE-mediated transcription by SU5416 is AHR dependent. A. The AHR-mutant C35 cell line was transfected with the AHR^b^, *lacZ* gene and a 3×DRE-Luc construct. Controls were transfected with the empty pSPORT vector plus the reporter constructs. After 24 h, the cells were treated with 3 µM SU5416 or 0.3% (v/v) DMSO, then incubated for 18 more h. Induction of AHR activity was determined by normalizing the luciferase activity to β-galactosidase activity. *White bars*: Empty vector. *Grey bars*: AHR. *Error bars*: SD; (n = 3). **B**. Induction of DRE-mediated transcription by SU5416 is ARNT dependent. The ARNT-deficient C4 cell line was transfected with the human ARNT or the pSPORT parent vector. These cells were also co-transfected, treated and assayed as in A. *White bars*: Empty vector. *Grey Bars*: ARNT. *Error bars*: SD; (n = 3). **C.** SU5416 is a ligand of the AHR. The hepatic cytosolic fraction from C57BL/6J mice was incubated with 1 nM of the radioligand ^125^BR_2_N_3_DpD, in the presence of increasing concentrations of competitor, SU5416, TCDD, BNF or 1,2-Benzanthracene. Ordinate: Specifically bound radioligand in the presence of competitor divided by specifically bound radioligand in the absence of competitor. Abscissa: The concentration of competing ligand, represented as log of molar concentration. Each data point represents the average of two determinations. Competitive binding to the C57BL/6J cytosol produced the IC_50_ values of SU5416 = 2.1 nM, TCDD = 1.5 nM, BNF = 2.8 nM, and 1,2-Benzanthracene = 13.7 nM.

To prove that induction of the DRE was mediated through classic AHR signal transduction, and not through a VEGF-related mechanism, we employed mutant cell lines that lack expression of the AHR or ARNT. The C35 cell line, which contains a dysfunctional AHR, was utilized [18]. It was transfected with vector containing the murine AHR gene, the *lac*Z gene, and the luciferase reporter gene driven by 3 upstream DREs, as described in the Methods section. Controls were mock transfected with reporter plasmids and the empty vector. Cells were treated with either 3 µM SU5416 or DMSO (control). As seen in figure 2A, cells transfected with the AHR plasmid generated significant luciferase activity when exposed to SU5416 compared to DMSO. The control cells generated minimal activity.

In a similar experiment, the ARNT-deficient mouse hepatoma cell line C4 was transiently transfected with plasmids encoding human ARNT, the *lac*Z gene, and the same DRE-driven luciferase gene, and control samples received empty vectors for ARNT [19], [20]. As shown in figure 2B, after exposure to SU5416 or DMSO, activity was only seen when ARNT was transfected.

### SU5416 is a Ligand of the AHR

To confirm that this molecule is a direct ligand of the AHR and not working through some other agonist, we performed competitive binding assays of the AHR using a radioligand. Photoaffinity experiments incubating ^125^IBr_2_N_3_DpD with the hepatic cytosolic fraction from C57BL/6J mice (AHR^b^ isoform) were conducted as described in the Methods [21]. Increasing concentrations of SU5416, TCDD, BNF, and 1,2-Benzanthracene (a ligand of low receptor affinity) were added. As shown in figure 2C, SU5416 competitively displaced the radiolabel with efficacy similar to TCDD.

### *In utero* Exposure to SU5416 Stimulates Closure of DV

We have previously shown that genetically altered mice that express only 10% of the AHR display a patent ductus venosus (DV) in the liver in nearly all cases [22]. We additionally identified that *in utero* activation of the receptor in the hypomorphs with TCDD successfully closed the DV [5]. To test the role of SU5416 as an *in vivo* ligand and its potential effect on embryology and vascular development, we performed timed matings of female AHR^fxneo/+^ mice to male AHR^fxneo/fxneo^ mice. The pregnant dams were treated at embryonic day E18.5 with a single dose of SU5416 at 110 mg/kg, or an equivalent volume of the vehicle, corn oil. At 4 weeks of age, the pups were sacrificed, and DV status was examined by hepatic perfusion with trypan blue. As seen in Table 1, only 1 of 25 AHR^fxneo/fxneo^ pups treated with corn oil possessed a closed DV. In the experimental group, 13 of 22 animals of this phenotype exposed to SU5416 had a closed DV.

**Table 1:** *In utero* exposure to SU-5416 stimulates closure of DV.

Pup genotype	SU5416	Corn Oil
AHR^fxneo/fxneo^	59% (13/22)	0.04% (1/25)
AHR^fxneo/+^	100% (31/31)	100% (34/34)

### SU5416 Upregulates CYP1A1 and CYP1B1

**Figure 3:**
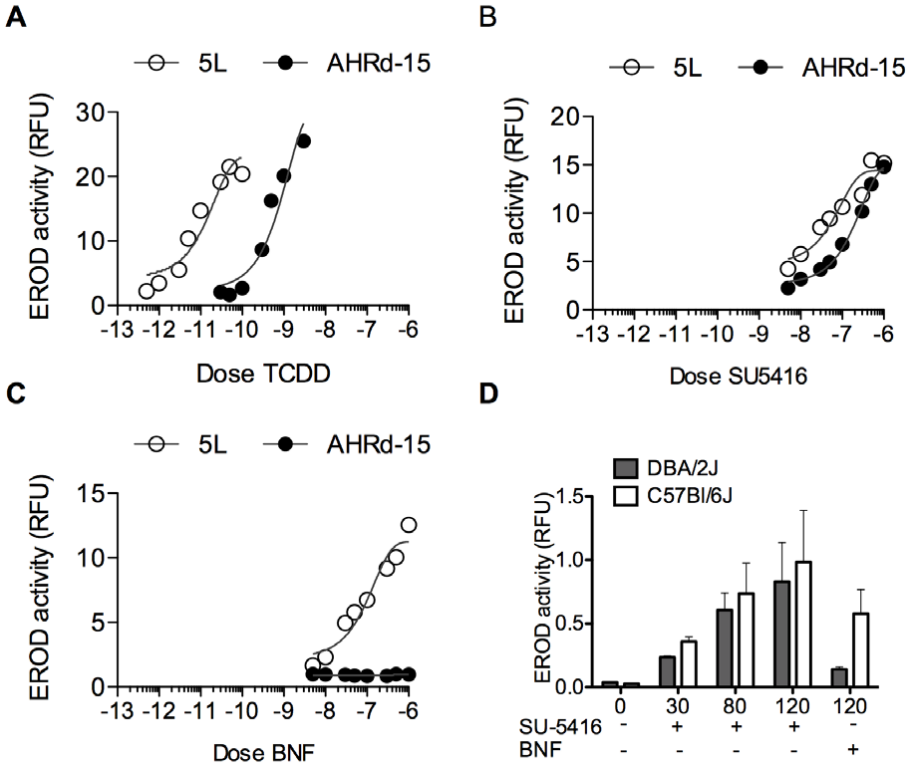
*In Vitro* dose response curves. A. Rat hepatoma cell lines bearing the murine AHR^d^ (AHR^d^-15) or the rat AHR^b^ (5L) were incubated with increasing doses of TCDD in 96-well plates. After 40 hours, EROD activity was performed from whole cell lysate. (n = 3). **B.** Same assay as in A, with increasing doses of SU5416. (n = 3). **C.** Same assay as in A, with increasing doses of BNF. (n = 2). **D.** Male DBA/2J and C57BL/6J mice (6 weeks old) were orally administered 120 mg/kg BNF, the indicated doses of SU5416 or the vehicle corn oil. Following 48 hours, hepatic microsomal proteins were isolated and used for the assessment of EROD activity. Animals were treated in groups of 4. *Error bars:* S.D.

The above data clearly shows that SU5416 is a ligand of the AHR. We now focused our attention on the strong response of SU5416 to the AHR^d^ polymorphism in the screening assay, and compared the activity of this ligand in the high and low affinity polymorphisms. We utilized the wild type rat hepatoma cell line, 5L, which harbors the high affinity AHR isoform, and our newly created AHR^d^-15 cell line. As seen in figure 3A, we first performed a titration with TCDD and measured EROD activity. As expected, the activity of TCDD was shifted by 1.5 orders of magnitude to the left for the AHR^b^ isoform. In contrast, when SU5416 was tested *in vitro*, the two curves virtually overlapped (figure 3B), showing equal potency for cytochrome P450 induction using the two cell lines. We also tested BNF, which as expected, showed a strong response with the 5L cell line and no response with the AHR^d^-15 cell line (figure 3C). As these experiments were done in cell lines, and in addition the AHR^d^-15 line combines a rat cell line with a transfected murine AHR, we further tested the ability of SU5416 to activate the AHR *in vivo*. Six-week old C57BL/6J mice (AHR^b^) and DBA/2J (AHR^d^) were orally administered 30, 80, or 120 mg of SU5416 per kg of body weight. Groups of control mice were given corn oil alone or BNF at the concentration of 120 mg/kg. Treatment of SU5416 produced a dose-dependent increase in EROD activity in both strains of mice seen after sacrifice (figure 3D), with no significant difference between the two. BNF at 120 mg/kg showed dramatically decreased *in vivo* activity in the DBA/2J strain. To identify the duration of activity of SU5416 as a ligand of the AHR, we dosed human 101L-hepatoma cells with SU5416 at a dose of 100 nM or TCDD at 1 nM, and measured luciferase activity at 4, 24, 48, 72, and 96 hours. As can be seen in figure S2A, SU5416 has activity at 4 hours, but by 24 hours no longer causes luciferase activity indicating loss of binding to the DRE, which is clearly in contrast to the long duration DRE-binding seen with TCDD. Of note, when we did titrate SU5416 doses as high as 10 µM, we did observe as much as 20% of TCDD response (1 nM) as far out as 96 hours (data not shown). This SU5416 data is similar to the known plasma half-life of 30 minutes, although VEGF-receptor inhibitor effects have been shown to last as much as 72 hours in culture [23]. We further analyzed whether the AHR antagonist CH223191 could inhibit the ability of SU5416 to activate the DRE in 101L-hepatoma cells. It has previously been shown that this antagonist inhibits TCDD but not some of the other ligands of the AHR including some polycyclic aromatic hydrocarbons. We first performed a titration of the AHR antagonist in culture with either 1 nM TCDD or 100 nM SU5416. As can be seen in figure S2B, the effects of TCDD are inhibited whereas minimal inhibition is shown for SU5416. In figure S2C, we show a titration of SU5416 with only a small amount of inhibition of activity by the antagonist (10 µM).

### SU5416-induced Upregulation of CYP1A1 is Similar in Murine AHR^b^ and AHR^d^ Splenocytes

**Figure 4:**
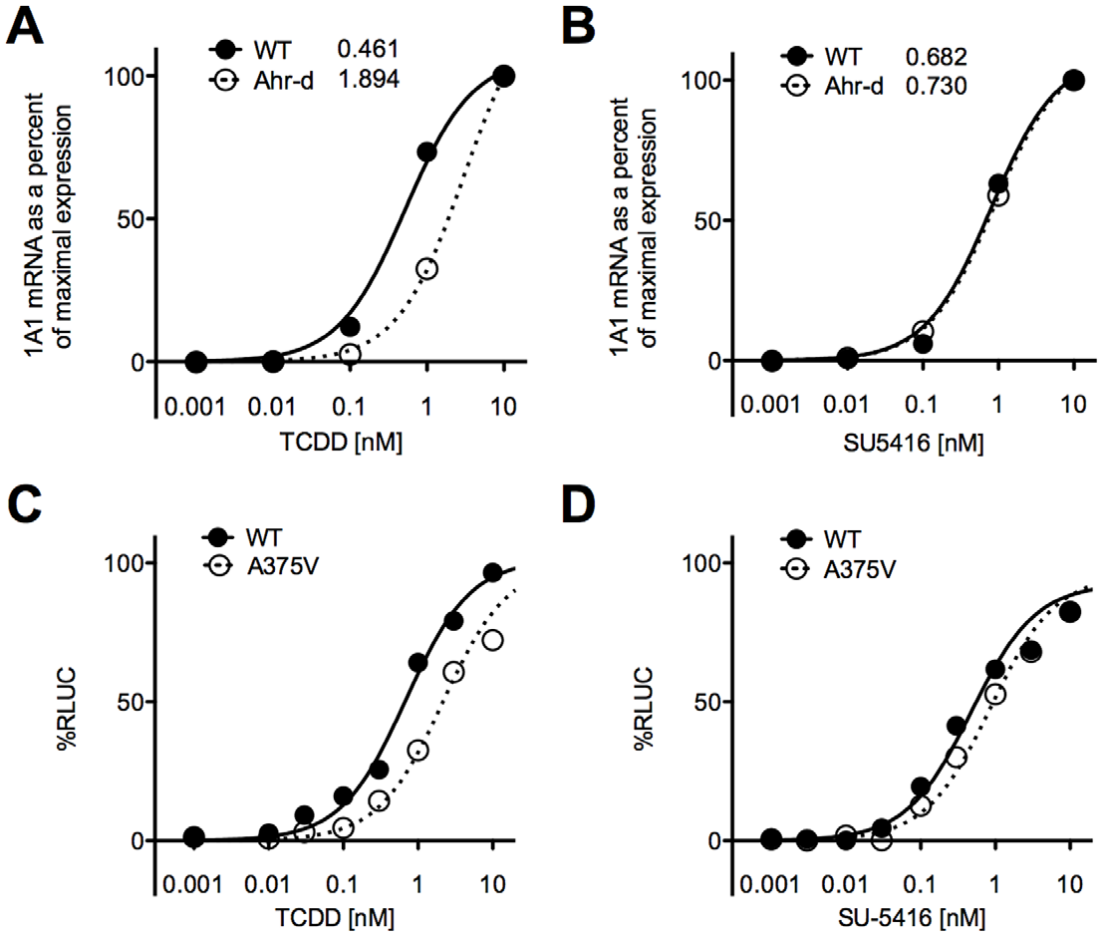
Splenocytes from wild-type and AHR^d^ mice analyzed by qPCR for CYP1A1. Spleens from these mice were harvested and suspended in culture media, and exposed to titrating doses of **A)** TCDD **B)** SU5416. After 4 hours they were analyzed by qPCR for CYP1A1 analysis. The curves are normalized from 0 to 100% response. Each graph is representative of 3 independent experiments. **C–D.** Cells transfected with AHR containing a valine point-mutation show similar ED_50_ to Cos-1 cells with AHR^b^ isoform. Cos-1 cells were transfected with an AHR containing the same point mutation (valine for alanine) thought to be responsible for the low affinity of the AHR^d^ isoform compared to AHR^b^, and compared to the wild-type AHR response. These cells also harbor a luciferase gene next to the DRE. **C.** Cos-1 cells were exposed to TCDD. **D.** Cos-1 cells were exposed to SU5416. The graphs represent normalized data from 0 response to 100% response. They are representative of 2 independent experiments.

As the above *in vitro* experiments were performed in cell lines, we next utilized AHR^b^ (C57BL/6J) and AHR^d^ congenic mice (on a C57BL/6J background). Spleens from these mice were harvested and suspended in culture media, and exposed to titrating doses of TCDD and SU5416. These data are presented in figure 4, where the graphs show normalized data from 0 to 100% response. Normalized data was chosen to allow comparison of CYP1A1 upregulation to its maximum in AHR^b^ versus AHR^d^ mice. After 4 hours of culture, TCDD induced CYP1A1 more rapidly and to a higher degree in wild-type than AHR^d^ splenocytes, with an EC_50_ of 0.461 nM in wild-type and 1.894 nM in AHR^d^ animals. Figure 4B shows that SU5416 induced CYP1a1 similarly in AHR^b^ and AHR^d^ mice, with an EC_50_ of 0.682 nM in wild-type and 0.730 nM in AHR^d^ mice. Figures S3A and B show the total fold change seen by qPCR analysis of splenocytes after exposure to TCDD and SU5416, to allow an assessment of the potency of AHR activation of these two ligands with CYP1A1 induction as the readout. As can be seen in the figure, TCDD elicits more CYP1A1 in AHR^b^ compared to AHR^d^ mice, whereas SU5416 leads to the same or more CYP1A1 in AHR^d^ mice. By this readout, TCDD and SU5416 have similar potency in AHR^d^ cells, and TCDD is a stronger ligand in AHR^b^ cells.

### Cos-1 Cells Transfected with AHR Containing Valine Point-mutation Show Similar ED_50_ to Cos-1 Cells with AHR^b^ Isoform

Because of the importance of identifying that SU5416 is truly unique in its ability to activate the low affinity AHR isoform with similar strength as the high affinity isoform, we transfected COS-1 cells with an AHR containing the same point mutation (valine for alanine) thought to be responsible for the low affinity of the AHR^d^ isoform compared to AHR^b^. These cells also harbor a luciferase gene next to the DRE. We again tested SU5416 and TCDD, harvesting the cells after 4 hours. As seen in figure 4C, the ED_50_ for TCDD was higher in the A375V transfected cell line (AHR^d^ type) compared to wild-type, with a much smaller difference between the two isoforms for SU5416 (figure 4D). Specifically, the EC_50_ for TCDD was 0.73 nM in wild-type cells, and 2.47 nM in transfected cells, while for SU5416 the EC_50_ was 0.18 nM in wild-type, and 0.31 nM in transfected cells. The EC_50_ is actually lower for SU5416 than TCDD in either cell line, supporting that this is a potent and unique ligand of the AHR that has only mild loss of binding capacity when faced with the valine point mutation see in the AHR^d^ isoform. The data in this figure is normalized from 0 to 100% response. The actual luciferase values are included in figures S3C and D, which again show the potency of these two ligands.

### SU5416 Leads to IDO Induction and FoxP3 Upregulation in CD4^+^ T Cells

**Figure 5:**
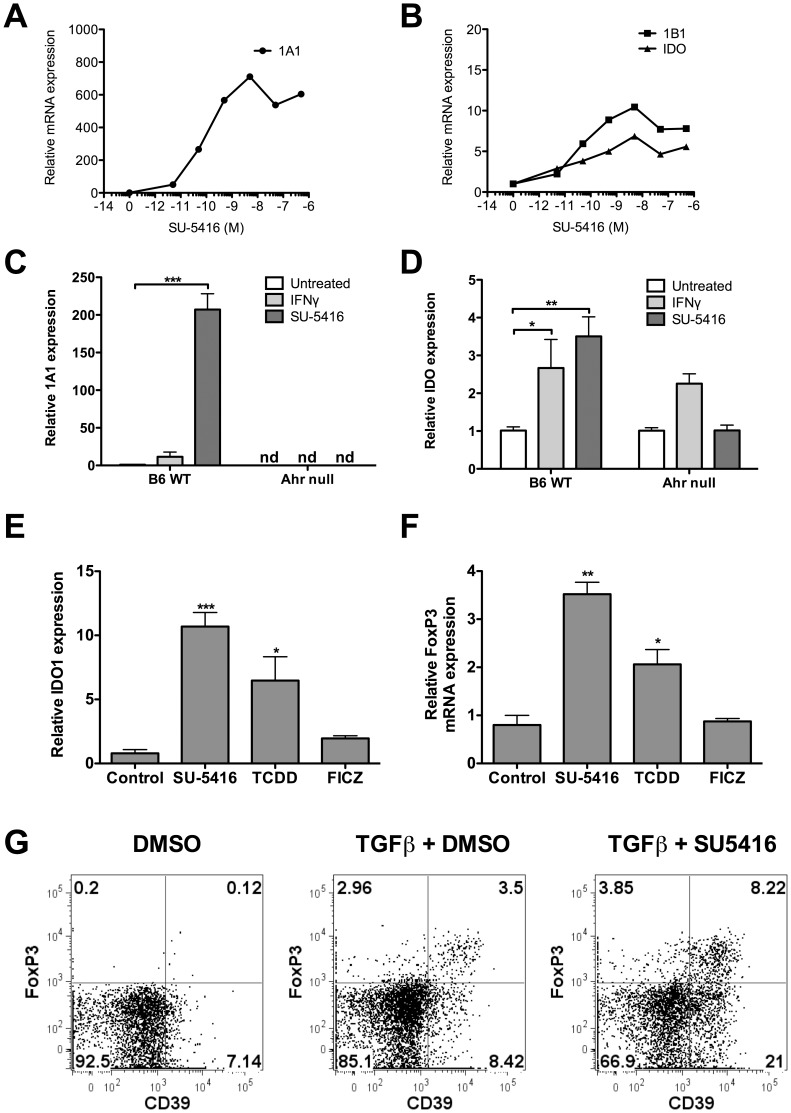
SU5416 upregulates CYP1A1. **A.** Spleens were harvested from mice and processed in the standard fashion. Cells were incubated for 4 hours in culture with titrating doses of SU5416 as indicated, and afterwards mRNA was measured for CYP1A1. **B.** SU5416 upregulates CYP1B1 and IDO. Same assay as A, but mRNA for CYP1B1 and IDO were measured. **C.** Upregulation of CYP1A1 mRNA is dependent on the AHR. Splenocytes from C57BL/6J or AHR^−/−^ mice were exposed to media, IFN-γ 100 ng/ml, or SU5416 500 nM for 4 hours. mRNA was then harvested and assayed for CYP1A1. nd represents “not detected”. *** - p≤0.001. **D.** IDO upregulation by SU5416 is AHR-dependent. Same assay as in C, but IDO mRNA was assessed. * - p≤0.05. * - p≤0.01. **E.** SU5416 induces IDO in the pDC/T cell coculture to a greater extent than TCDD. pDC/T cell coculture was utilized as described previously [25]. Culture was performed for 5 days with SU5416 500 nM, TCDD 10 nM, FICZ 100 nM, or control, at which point mRNA was harvested and measured for IDO. * - p≤0.05. *** - p≤0.001. **F.** SU5416 induces FoxP3 in the pDC/T cell coculture to a greater extent than TCDD. Same assay as in E, but mRNA was assayed for FoxP3. * - p≤0.05. ** - p≤0.01. **G.** SU5416 enhances FoxP3 expression and CD39 on naïve T cells in the presence of TGF-β. Naïve T cells were placed in culture with DMSO (1∶4×10^4^ dilution), TGF-β (2 ng/ml), or TGF-β (2 ng/ml) and SU5416 (250 nM), harvested on day 3, and analyzed by flow cytometry. All of the above figures are representative of 3 independent experiments.

An important role for the AHR in the immune system, and specifically T-cell differentiation, has been recognized and continues to be characterized in the literature [6], [7]. Some ligands of the AHR have the ability to enhance Treg differentiation from naïve T-cells (TCDD, kynurenine), while others direct differentiation towards Th17 effector cells (FICZ). We first tested the ability of SU5416 to induce CYP1A1 and CYP1B1 when titrated in solution with cultured splenocytes. Spleens from C57BL/6J mice were harvested and suspended in culture media, and exposed to titrating doses of SU5416. As seen in figure 5A, after 4 hours of culture SU5416 dramatically induced these cytochrome P450 enzymes in a dose-dependent manner, indicating activation of the DRE *in vitro*. In this same assay we tested the ability of SU5416 to generate the CYP1B1 and the enzyme IDO, the first enzyme in the kynurenine pathway of tryptophan metabolism. IDO has long been known to play a role in Treg generation, and may be central to the mechanism of Dendritic Cell (DC)-directed Treg generation [24]. We as well as others have previously shown that IDO mRNA can be induced by ligands of the AHR, and that the mechanisms of IDO-directed Treg generation may depend on the AHR [25]. This assay shows that SU5416 induced significant amounts of IDO mRNA in splenocytes (figure 5B), a finding that was previously reported for TCDD [26]. To confirm that CYP1A1 and IDO induction in splenocytes is in response to an interaction with the AHR and not secondary to an interaction with the VEGF receptor, we compared the response of AHR wild-type and AHR^−/−^ cells to SU5416 and IFN-γ. As shown in figure 5C and D, SU5416 induced CYP1A1 in wild-type but not null cells. Additionally, IDO was induced by SU5416 in wild-type but not null cells, confirming the importance of this receptor in IDO induction. IFN-γ did lead to some IDO induction in both wild-type and null cells (although it did not reach statistical significance in the null cells in this representative assay), a finding we have seen in prior experiments [25]. To assess if FoxP3 could be generated by SU5416 exposure, we employed a pDC/T cell coculture. Previous authors have suggested that Treg generation in this assay is driven by IDO production by the plasmacytoid DCs (pDCs) [27]. As described in the Methods, naïve T-cells were sorted using magnetic bead separation, and placed in culture for 5 days with allogeneic pDCs separated from BALB/C mice. SU5416, TCDD, FICZ, or media alone was added at the start of culture. After 5 days, cells were collected and mRNA harvested for qPCR analysis of IDO and FoxP3. As shown in figure 5E and F, IDO and FoxP3 were generated after addition of SU5416 in this assay. This upregulation was also seen with TCDD, which has been previously reported to induce FoxP3 [6]. In order to look at the direct effect of SU5416 on T cells alone, we separated naïve CD4^+^ T cells and exposed them to TGF-β with or without SU516. We used a dose of 2 ng/ml TGF-β, which in our hands has been a suboptimal dose for Treg generation (4 ng/ml has been optimal in our hands). As can be seen in figure 5G, the addition of SU5416 significantly enhanced the FoxP3 protein expression by flow cytometry. To further support that SU5416 leads to regulatory cells, we also analyzed the upregulation of CD39, which is an ectoenzyme that degrades ATP to AMP and is strongly associated with Tregs that can suppress ATP-related effects and pathogenic Th17 cells. As can be seen in figure 5G, SU5416 upregulated CD39 in the FoxP3^+^ T cells, a finding that has recently been reported with TCDD [28]. Finally, as literature is emerging that the ability of AHR ligands to enhance T-cell differentiation may be dependent as much on surrounding conditions and inflammatory milieu as on the ligand tested, we assessed the ability of SU5416 to enhance Th17 differentiation in Th17 conditions. Naïve T cells were placed in culture with IL-6 and TGF-β, and harvested after 3 days of culture. Figure S4 shows that at low doses SU5416 caused a small increase in IL-17 protein by ELISA in the supernatant. At higher doses we did not see this effect.

## Discussion

SU5416 was specifically designed as an inhibitor to VEGF-R2 [29], with the hope that it would join the armamentarium of anti-angiogenesis drugs used to combat malignancy. It initially showed promise in preclinical trials, was used in phase I and phase II studies of hematologic and solid cancers [30], [31], [32], and ultimately did make it to phase III trials for metastatic colorectal cancer. While the drug was well tolerated, its ability to significantly reduce the growth of cancer over standard regimens was unimpressive, and it remains an experimental drug. While its clinical role remains unclear, SU5416 continues to be utilized in laboratory studies to confirm the importance of VEGF in various mechanistic studies, including cell trafficking, organ rejection, and autoimmunity [33], [34], [35].

The data presented in this manuscript demonstrate that SU5416 is a strong ligand of the AHR. The unique finding that SU5416 binds the high- and low-affinity polymorphisms of the AHR similarly was rather surprising to us, and will require further attention and characterization. The mouse AHR can arise from an allele that encodes a receptor with high binding affinity for ligand (denoted *Ahr^b^* allele) or with low binding affinity for ligand (denoted as the *Ahr^d^* allele). The AHR^d^ is known to have approximately one-fifteenth to one-twentieth the binding affinity to TCDD as the AHR^b^ [36], and this low affinity polymorphism resembles the isoform found in humans [37], [38], [39], [40]. C57BL/6 mice harbor the high-affinity AHR^b^ receptor, and this strain has been utilized for much of the initial characterization of TCDD and other environmental toxicants [3]. In our search for relevant ligands of the AHR, we decided to focus on those that had significant potency in the AHR^d^ isoform, as these ligands would have more clinical relevance in humans. We inadvertently identified that SU5416 had similar binding characteristics with both polymorphisms at doses that are similar to what were used in humans in Phase I trials with SU5416 [30], as seen in the titration in figure 3D. This is an unusual characteristic that has rarely been exhibited by any of the known ligands of the AHR [41]. The importance of this is due to the following: First, the information is clinically significant given that humans harbor an AHR isoform that more similarly represents the AHR^d^. Second, its structure will serve as a model in our search for endogenous ligands of the AHR. It makes sense that a true endogenous ligand would activate both polymorphisms of the AHR similarly, given that mice (and humans) that harbor the low affinity polymorphism do not exhibit the patent ductus venosus found in AHR nulls and hypomorphs. This is further supported by the ability of SU5416 to close the DV in AHR hypomorphs (a requirement of the true endogenous ligand) [12]. To this point we have been unable to model the binding sites of these polymorphisms by crystallography, but the finding that SU5416 can bind both of these similarly may help us in these efforts. At the very least, it confirms that a potential endogenous ligand that binds both isoforms equally might exist.

Ever since it was reported that some ligands of the AHR favor Treg generation and others favor Th17 differentiation, we have been categorizing novel ligands for their properties in T-cell differentiation. The above data support that SU5416 enhances Treg generation *in vitro*, and that IDO is generated in pDCs in response to SU5416 *in vitro* in an AHR-dependent manner. We continue to characterize these effects for multiple ligands, and are considering theories explaining these differences including the potency and duration of binding of the ligands to the receptor, a possible change in conformation of the receptor when different ligands bind, and a possible effect on APC-T cell interactions. That being said, there is some data to suggest that these dichotomous findings are not as clear cut as originally thought. Most of the *in vitro* studies examining effects on T-cell differentiation are done either in Treg or Th17 conditions, which are artificial by design. In addition it has been shown that FICZ, the ligand best associated with Th17 differentiation, can enhance Treg differentiation in the presence of TGF-β, and TCDD can enhance Th17 differentiation [42], [43]. This is similar to the data we show in supplementary figure 4, where SU5416 increases IL-17 in the supernatant of T cells cultured in Th17 conditions at low doses. It is likely that these effects are highly dependent on the ligand, the inflammatory milieu that is present in the assay or disease process, and the particular *in vivo* model system being studied. The prototypical regulatory ligand is TCDD, although others have been identified (kynurenine [25], ITE [44], VAF347 [8]). FICZ remains the most well characterized effector ligand. By further delineating the properties of these ligands and the inflammatory milieu that allow them to have disparate effects on T-cell differentiation, it may ultimately be possible to utilize these properties to treat various diseases. This will require more characterization *in vitro* and *in vivo*. We do not believe the ligand activity is attributed to an indirect effect driven by VEGF, due to the impressive and rapid competitive binding in the radioligand assay, and additionally because we did test other known inhibitors of VEGFR-2, and did not find consistent DRE-luciferase activity in the range of their activity with VEGFR-2 (VegFR-2 IC50 values were in the nanomolar range, while AHR activity was in the micromolar range or not active) (Figure S5). In addition to and independent of its effect on the AHR, SU5416 is certainly an inhibitor of VEGFR-2, as was well proven in previous studies [45]. The implications of our findings are important both for potential utility of this drug in humans, but also for mechanistic interpretations of previous experiments *in vitro* and *in vivo*.

Regarding previous *in vitro* and *in vivo* studies, there is strong data supporting a role for VEGF in immune cell migration and chemotaxis, generation of inflammatory cytokines, and angiogenesis. With that said, there are numerous studies that utilize SU5416 in experimental models and interpret the results based on its VEGF effect. For example, one recent paper analyzed the role of VEGF in airway inflammation *in vitro* and in a murine model [33]. The authors found that SU5416 blocked LPS-induced airway inflammation, and specifically the differentiation of T cells to Th17 cells, along with a reduction of IL-6. These data would be fully consistent with regulatory effects of the drug through the AHR (this exact effect has been shown to be AHR-dependent when driven by TCDD and kynurenine [6], [25]). While VEGF may also have a role in this differentiation, these data need to be interpreted carefully. In another study, daily injection of SU5416 is found to abrogate EAE in comparison to standard EAE induction with MOG peptide, which is presumed to be due to disruption of the effects of VEGF in this model [34]. Again, while it is possible that VEGF plays a role in EAE, these findings are identical to the results exhibited when animals in this protocol were treated with TCDD [6], [7], which is AHR-dependent. Other studies have similarly used SU5416 to demonstrate the importance of VEGF in cell trafficking [35], although there does appear to be a role for VEGF in this mechanism shown with experiments that didn’t involve SU5416. These are only a few of the hundreds of studies utilizing SU5416 to assess the importance of VEGF in various biologic mechanisms, as this has become a standard technique in experimental studies. While we are not asserting that VEGF is not involved in any of the above findings, consideration for a role of the AHR needs to be given.

SU5416 has demonstrated limited efficacy in human studies in its ability to affect cancer outcomes to this point, whereas some other pharmaceuticals targeting VEGF have enjoyed more success [46]. It is possible that the effects via the AHR, including IDO induction and Treg generation actually outweigh some of the anti-cancer effects of the drug, as it is postulated that cancer cells utilize IDO and its regulation to prevent their destruction by immune mediators of tumor surveillance [47]. A recent paper highlighted the point that human brain tumors promote tumor progression by activation of IDO and the kynurenine pathway, which is likely dependent on Treg generation [48]. Another concern about using this drug in combination cancer therapy is that like other ligands of the AHR, it does induce cytochrome P450 enzymes, which can cause its own metabolism as well as that of other coadministered pharmaceuticals. Careful attention needs to be directed at the metabolism of drugs used together with SU5416. These characteristics may explain the disappointing results with this drug in clinical trials in contrast to other related compounds [49].

Perhaps equally important and exciting is the potential for this drug, already found to be safe in humans, to have multiple mechanisms that could be beneficial for treatment of diseases not yet considered. Two areas where we speculate that there could be potential are in autoimmunity and transplant rejection. While angiogenesis, stimulated by VEGF and other factors, can have a protective and regenerative role in response to tissue injury, it has also been linked to chronic inflammation, fibrosis, and tissue injury in both preclinical models and in human autoimmune diseases, including systemic lupus erythematosus, rheumatoid arthritis, vasculitis, multiple sclerosis, and asthma, to name a few [50]. Additionally VEGF may play a role in acute and chronic rejection, with copious amounts of this growth factor released by immune cells leading over time to fibrosis and ultimately organ failure [51]. These data have made VEGF and its receptors an enticing target for future intervention in these disease processes. At the same time, we have already discussed a role for the AHR in the pathogenesis of both autoimmunity and organ rejection. We have a recent publication where ligands of the AHR can both inhibit, or alternatively accelerate rejection of skin grafts in fully mismatched mice, depending on the ligand utilized [52]. Another study shows the ability of a ligand to promote tolerance to islet cell transplantation across a full MHC mismatch in mice [53]. These data would support the efficacy of a drug with these properties for treatment of autoimmunity and transplant rejection. There are already a few approved pharmaceuticals that likely function via the AHR (including treatments for asthma and organ rejection) [54], but none that combines the effect of VEGF blockade with modulation of the AHR. This could represent a novel angle to improve understanding of the mechanisms behind autoimmunity and organ rejection, and will provide a new class of drugs to combat these debilitating diseases.

## Materials and Methods

### Animals

Five to twelve week old male C57BL/6J, DBA/2J, and BALB/cJ mice were obtained from The Jackson Laboratory (Bar Harbor, ME). AHR-deficient mice on a C57BL/6J background (AHR^−/−^) were bred and maintained under specific pathogen-free conditions [55]. This study was carried out in strict accordance with the recommendations in the Guide for the Care and Use of Laboratory Animals of the National Institutes of Health. All animal experiments were carried out according to institutional guidelines with appropriate IRB approval from the University of Wisconsin-Madison Animal Care and Use Committee, under protocol number M02293-0-09-11.

### Cell Culture

All cell lines and isolated cell preps were cultured in Dulbecco’s modified medium with high glucose supplemented with 10% (v/v) fetal bovine serum, 1 mM sodium pyruvate, 2 mM L-glutamine, 10 µM HEPES buffer solution, 0.1 mM minimal essential medium nonessential amino acids, and penicillin-streptomycin at 100 U/ml and 100 µg/mL, respectively (all media reagents from Invitrogen Corp., Carlsbad, CA). Cell cultures were maintained in a standard 5% CO_2_, 37°C environment. The 101L cell line harbors a stably transfected luciferase reporter driven by 3 upstream DREs [13]. The mouse hepatoma cell line C4 lacks expression of ARNT while the C35 cell line expresses a mutant AHR incapable of nuclear translocation and binding DRE [18], [19], [56], [57]. The C4 and C35 cell lines were provided by Dr. Oliver Hankinson (Los Angeles, CA) [18], [19] and the 101L cell line was a gift of Dr. Robert Tukey (San Diego, CA) [13].

### Small Molecule Screen

A library of 4160 small molecules, designated the “Known Bioactive Library”, was screened for inducers of AHR at the Small Molecule Screening Facility (University of Wisconsin, School of Medicine and Public Health, Madison, WI). This includes 2,000 diverse FDA approved drugs and natural products (Microsource Discovery Systems, Inc; Gaylordsville, CT); the 1280 compound LOPAC^1280^ library of diverse characterized compounds (Sigma; St Louis, MO); and 880 characterized compounds (Prestwick Chemicals; Illkirch, FR). Further details can be obtained at http://hts.wisc.edu/htslibraries.php. The compounds were dissolved in DMSO to generate 1 mM stocks and were arrayed in 384-well plates. High-throughput compound screening was performed in 384-well plates using the 101L reporter cell line and the Biomek automated liquid-handler (Beckman Coulter, Inc., Fullerton, CA). To each well, 100 µL of culture media containing ∼ 8000 cells and 1 µL test compound were added (1% DMSO, v/v). At ∼ 24 hours post-treatment, luciferase activity was assayed by first removing 60 µL of culture media from the wells and then adding 30 µL of Bright-Glo™ luciferase reagent to the cells (Promega, Madison, WI). Induction of luciferase activity was monitored in the Perkin-Elmer Victor 3-V microplate luminometer (Waltman, MA). Basal luciferase signal produced from cells treated with DMSO alone served as controls from which the fold-induction of AHR activity was calculated.

### Generation of AHR^d^-15 Cell Line

The open reading frame of the AHR^d^ gene (PL2047) was amplified by PCR using oligos OL5127 (5′-GAACCATGAGCAGCGGCGCC-3′) and OL5128 (5′-CCCTACAGGAATCCACCAGGTGTGATATC-3′). The product was ligated into the pTARGET™ mammalian expression vector (Promega, Madison, WI). The resulting plasmid was transfected into the AHR-deficient cell line, BP8 [15], using Effectene transfection reagent (Qiagen Inc., Valencia, CA). Stable integrants were selected with 6 mg/ml of G418 for 2 weeks, beginning at 48-hours post-transfection. Clones derived from single cells were screened for their response to nanomolar doses of dioxin or micromolar doses of BNF, as measured by ethoxyresorufin O-deethylase (EROD) activity. Confirmation of AHR^d^ expression in select cell lines was achieved by Western blot using the BEAR-3 anti-AHR antibody that recognizes the PAS domain [58]. The validated cell line used in these studies was designated line AHR^d^-15.

### Transfection and Luciferase Assays

To determine ED_50_ for TCDD or SU5416-induced AHR activity, COS-1 cells [59], [60] (Sigma; St Louis, MO) were transiently transfected with pTarget-mAHR or pTarget-mAHR-A375V expression plasmid, pGudLuc6.1 luciferase reporter plasmid and TK-renilla luciferase plasmid using Lipofectamine™ 2000 (Invitrogen, Carlsbad CA). Six hours after transfection, cells were cultured with DMEM media containing TCDD (1×10^−7^–1×10^−13 ^M), SU5416 (3×10^−7^–1×10^−12 ^M) or vehicle alone (0.1% DMSO). After 4 hours of treatment, cells were assayed with dual luciferase® reporter assay system (Promega, Madison, WI). The expressed luciferase activity was measured by MicroLumat Plus luminometer (Berthold Technologies, Hartfordshire, UK). The dose-response curves and ED_50_ values for TCDD and SU5416 were determined using GraphPad Prism 4 software (GraphPad software Inc., La Jolla, CA).

For other luciferase assays, a mouse hepatoma cell line H1L6.1c3, stably carrying a dioxin-responsive element (DRE)-driven firefly luciferase reporter gene [a gift from Dr. Denison, University of California, Davis, CA [61]] was maintained with 0.3mg/ml G418 in completed DMEM media. Briefly, 0.6×10^6^ cells were seeded in each well of a six-well plate overnight and were then treated with SU5416 or other ligands at the dose described in the text. Cells were lysed by lysis buffer (Promega, Madison, WI), and the luciferase assay was performed by using a BD moonlight 3010 luminometer (BD Biosciences, San Jose, CA). The relative light unit is the indicator of luciferase expression level.

### Validation of SU5416

To assess the role of the AHR in SU5416-induced DRE-dependent gene transcription, the C35 AHR mutant cell line was transiently transfected with an expression vector containing the AHR^b-1^ cDNA (PL65), the pCH110 *lacZ* plasmid, and a vector containing a DRE-luciferase construct (PL265)., Control samples were mock transfected with the two reporter plasmids and the empty pSPORT vector (PL22), the parent vector from which PL65 was derived. The cells were seeded into 24-well plates at ∼ 60% density and transfected with 67 ng of each plasmid DNA. Following 24 hours, 3 µM SU5416, 3 µM BNF or 0.3% (v/v) DMSO was added. The cells were cultured for an additional 18 hours prior to assessment of luciferase and β-galactoside activity using commercial kits (Promega, Madison, WI). The ARNT-deficient cell line, C4, was transfected, treated, and assayed as described above for the C35 cells. However, in place of the AHR-bearing plasmid, these cells were transfected with the human ARNT (PL87), plus the luciferase and *lacZ* reporter plasmids.

### Isolation of Hepatic Microsomal Fraction

Hepatic microsomes were prepared by homogenizing 0.5 g of liver tissue in 5 mL of MENG buffer (25 mM buffer sodium morpholinopropane sulfonate buffer (pH 7.5) containing 0.025% (w/v) NaN_3_, 1 mM EGTA and 10% (v/v) glycerol). The homogenate was subjected to centrifugation at 10,000×g for 20 minutes, followed by centrifugation of the supernatant for 1 hour at 100,000×g and 4°C. The pellet was dissolved in 15 mM Tris-HCl buffer containing 250 mM sucrose (pH 8.0) and aliquots were stored at −80°C. Protein concentration was determined using the Bicinchoninic Acid Protein Assay Reagent (Pierce Biotechnology, Rockford, IL).

### High Throughput Ethoxyresorufin O-Deethylase (EROD) Analysis

High-throughput analysis of EROD activity was assayed by adapting a protocol that was described elsewhere [62], [63]. Briefly, cells were seeded into 96-well plates at ∼ 60% density and treated with the test compounds for 36 hours. The cells were washed with PBS and lysed with 30 µL water and a cycle of freeze-thaw. To each well, 150 µL of 50 mM Hepes buffer containing 26.7 µM dicumarol and 13.3 µM ethoxyresorufin were added. The samples were incubated at 30°C for 20 minutes, and 50 µL of 0.5 mM β-NADPH were added to initiate the reaction. Fluorescence of the resorufin product was detected using the 544 nm excitation and 590 nm emission wavelength filter set.

### Dose-dependent Response to SU5416 in Mice

Five-week old male C57BL/6J and DBA/2J mice were obtained from Jackson Laboratories (Bar Harbor, ME) and housed at the University of Wisconsin animal facility. Groups of four mice were orally administered 30, 80, or 120 mg of SU5416 per kg of body weight. Control groups were dosed with BNF at 120 mg/kg or an equivalent volume of corn oil (30 mL/kg). After 48 hours, liver tissue was collected for preparation of microsomes and determination of EROD activity.

### Photoaffinity Ligand Binding

Hepatic cytosolic fractions were isolated from the livers of male C57BL/6J mice. Cytosolic fractions were diluted to 1 mg of protein per mL of 25 mM sodium morpholinopropane sulfonate buffer (pH 7.5) that contains 0.025% (w/v) NaN_3_, 1 mM EGTA, 10% (v/v) glycerol, 15 mM NaCl, 1.0 mM dithiothreitol and 0.10% (v/v) Nonidet NP-40. A 1 nM concentration of radioligand, [^125^I]2-azido-3-iodo-7,8-dibromodibenzo-*p*-dioxin (^125^IBr_2_N_3_DpD), was incubated with increasing concentrations of the competing compound [64]. The binding reactions were performed at 20^0^ C for 30 min, followed by incubation at 0°C for 5 min to minimize ligand dissociation. Charcoal and gelatin (respective concentration of 1% and 0.1%, w/v) were added for 10 min at 0°C to absorb the unbound ligand and then removed by centrifugation. The ^125^IBr_2_N_3_DpD –bound cytosolic fractions were UV-irradiated with four Photodyne 300 nm wavelength lamps at a distance of 4 cm for 1 minute. The protein was precipitated by an overnight incubation in acetone at −20°C, collected by centrifugation and washed with cold acetone:water (9∶1, v/v). The washed pellet was dissolved in sodium dodecyl sulfate sample buffer and resolved by electrophoresis on a 7.5% (w/v) polyacrylamide gel. Following staining and drying, the gel was exposed to film overnight at −80°C with an intensifying screen. The 95-kDa AHR^b-1^ band was excised and the amount of ^125^IBr_2_N_3_DpD covalently bound was quantified in a γ counter.

### Assessment of Ductus Venosus Status

Timed mating of female AHR^fxneo/+^ mice to male AHR^fxneo/fxneo^ mice was performed [5]. At gestation day E18.5, the pregnant dams were injected i.p. with 110 mg/kg of SU5416 or the vehicle, corn oil. When the pups were 4 weeks of age, the status of the DV was determined by hepatic perfusion with Trypan Blue as previously described [5]. Briefly, each mouse was anesthetized and its liver was flushed with PBS through the cannulated portal vein. The inferior vena cava was incised to allow outflow. Trypan blue was injected through the portal vein until the liver visibly turned blue (in the case of a closed DV) or until the dye was seen exiting the IVC without perfusing the liver (when the DV is open).

### Real-time Quantitative PCR (qPCR)

Spleen was harvested from mice at the time of euthanasia and single cell suspensions were made. RBCs were eliminated from spleen preps using RBC Lysing Solution (eBioscience). Total RNA was extracted using the reagents: RNeasy Mini Kit and RNase-Free DNase Set (Qiagen, Valencia, CA). A total of 500 ng total RNA in each group was used for RT reaction (iScript cDNA Synthesis Kit, Bio-Rad, Hercules, CA; or High-capacity cDNA Reverse Transcription Kits, Applied Biosystems, Foster City, CA). The relative quantitation PCR for IDO1 (Mm00492586-m1) and GAPDH (4352339-E0806018) were performed in the Applied Biosystems 7900HT Fast Real-Time PCR System (Applied Biosystems), and TaqMan Universal PCR Master Mix (Applied Biosystems) was used as a reaction reagent. The relative quantitation PCR for Foxp3, CYP1A1, CYP1B1, and GAPDH were processed by the Bio-Rad iCycler (Bio-Rad) and iQ SYBR Green Supermix (Applied Biosystems) was used as the reaction reagent.

### Isolation of Naive CD4^+^ T Cells and T Cell Differentiation

Naive CD4^+^ T cells were isolated using the CD4^+^ CD62L Isolation Kit (Miltenyi Biotec, Auburn, CA) and an autoMACS. This kit includes a depletion mixture, including the addition of a CD25 and an anti-TCRγ/δ^+^ Ab. Cells were tested for purity post-sorting and consistently showed >90% purity for CD4^+^CD62^+^CD25^−^ cells. Viability at the beginning of culture was typically >98% as seen by trypan blue staining. For quantitative PCR (qPCR) analysis, 2–5 × 10^5^ cells were cultured in each well of a 96- well round-bottom plate coated with 0.5 µg/ml anti-CD3 and anti-CD28 overnight and then washed with PBS twice before seeding the cells. The naive T cells were maintained in F10 media supplemented with 10% heat-inactivated FBS, 100 µg/ml streptomycin, 100 U/ml penicillin, 50 µm 2-ME, 25 mM HEPES, and 2 mM L-glutamine. Cells in Treg conditions included the addition of TGF-β (2 ng/ml). Th17 conditions include TGF-β (4 ng/ml) and IL-6 (20 ng/ml) for 3 days.

### pDC/T Cell Coculture

Naive CD4^+^CD25^−^ T cells were isolated from WT and AHR-null mice and cocultured with BALB/cJ pDCs isolated using the Miltenyi Mouse pDC Isolation Kit (Miltenyi Biotec) at a ratio of 20∶1 or 10∶1 as previously described (13). SU5416, TCDD (10nM), or FICZ (100 nM) were added at the start of culture. On day 5, cells were harvested and subjected to qPCR analysis.

### Flow Cytometry

To stain for FoxP3, T cells were first surface stained with anti-CD4 and antiCD25 and then fixed and permeabilized with the Fixation/Permeabilization buffer (eBioscience, San Diego, CA) for 30 min at 4°C. Following this, cells were stained with Pacific Blue-conjugated anti-FoxP3. For intracellular IL-17 staining, T cells were first stimulated with 50 ng/ml PMA (Signma-Aldrich) and 800 ng/ml ionomycin (Sigma-Aldrich) for 4 h in the presence of GolgiStop (BD Pharmingen, San Diego, CA) for the final 2 h. Cells were then fixed and permeabilized with the Fixation/Permeabilization buffer (eBioscience) and then stained with PE-conjugated anti-IL-17. All abs were from eBioscience. Flow cytometric analysis was performed using an LSR-II (BD Biosciences). CD39 antibodies for flow cytometry were purchased from eBioscience.

## Supporting Information

Figure S1 **Characterization of AHR^d^-15 cell line.** **A.** A Western blot was performed using the whole cell lysate of AHRd-15 cells. Lysate from the AHR null BP8 parental cell line, and the hepatic cytosolic fractions from C57BL/6J and DBA/2J mice were included as size controls. Proteins were resolved by electrophoresis on a 7.5% acrylamide gel, and then probed with the BEAR-3 anti-AHR antibody. **B.** AHR^d^-15 is responsive to TCDD, but not BNF. Dose-response curves were generated by treating AHR^d^-15 cells with nM doses of TCDD and µM doses of BNF for 36 hours. Activation of the AHR^d^ was determined by quantifying EROD activity from whole cell lysate.
(TIFF)

Figure S2 **Duration of action of AHR activation.** **A.** 0.6×10^6^ Cells from a mouse hepatoma cell line H1L6.1c3, stably carrying a dioxin-responsive element (DRE)-driven firefly luciferase reporter gene were seeded in each well of a six-well plate overnight and were then treated with SU5416 100 nM or TCDD 1 nM for 4 hours through 96 hours. DRE activity was assayed by a luminometer at the time points shown. Data is presented as a percent of 100 nM TCDD response at those time points. **B–C.** Characterization of antagonism of response of SU5416 by CH223191. **B.** SU5416 100 nM or TCDD 1 nM were tested with DRE-driven luciferase reporter cells with titrating doses of the antagonist for 4 hours as delineated in the figure. Data is presented as % response without inhibitor. **C.** SU5416 was titrated in culture with and without the antagonist (10 µM) for 4 hours. Results are presented as % maximal TCDD response at 100 nM.
(TIFF)

Figure S3 **Similar to** **figure 4** **, splenocytes from wild-type and AHR^d^ mice analyzed by qPCR for CYP1A1.** Spleens from these mice were harvested and suspended in culture media, and exposed to titrating doses of **A)** TCDD **B)** SU5416. After 4 hours they were analyzed by qPCR for CYP1A1 analysis. The curves represent fold change and show the similar potency of these ligands. Each graph is representative of 3 independent experiments. **C–D.** Cells transfected with AHR containing a valine point-mutation show similar ED_50_ to Cos-1 cells with AHR^b^ isoform. Cos-1 cells were transfected with an AHR containing the same point mutation (valine for alanine) thought to be responsible for the low affinity of the AHR^d^ isoform compared to AHR^b^, and compared to the wild-type AHR response. These cells also harbor a luciferase gene next to the DRE. **C.** Cos-1 cells were exposed to TCDD. **D.** Cos-1 cells were exposed to SU5416. The graphs represent true luciferase values. They are representative of 2 independent experiments.
(TIFF)

Figure S4 **SU5416 causes a small amount of IL-17 secretion at low doses.** Naïve T-cells were placed in Th17 conditions in culture (TGF-β 4 ng/ml, IL-6 20 ng/ml) and exposed to titrating doses of SU5416 as indicated. After 3 days of culture, supernatant was harvested and tested for IL-17 by ELISA.
(TIFF)

Figure S5 **Activation of the AHR by VEGFR-2 kinase inhibitors.** Different VEGFR-2 kinase inhibitors were tested for their ability to activate 101L cells in a luciferase assay, signifying AHR activity. Cells were incubated for 20 hours with mid-log concentrations of these compounds ranging from 0.01–30 µM. EC_50_ were calculated. These values were compared to reported IC_50_ of these compounds for inhibition of phosphorylation activity of VEGFR-2.
(DOCX)
