# Supplementary figures and images for: SU5416, a VEGF Receptor Inhibitor and Ligand of the AHR, Represents a New Alternative for Immunomodulation
Source: PLoS One. 2012 Sep 6;7(9):e44547. doi: 10.1371/journal.pone.0044547 (PMC3435281; doi:10.1371/journal.pone.0044547)

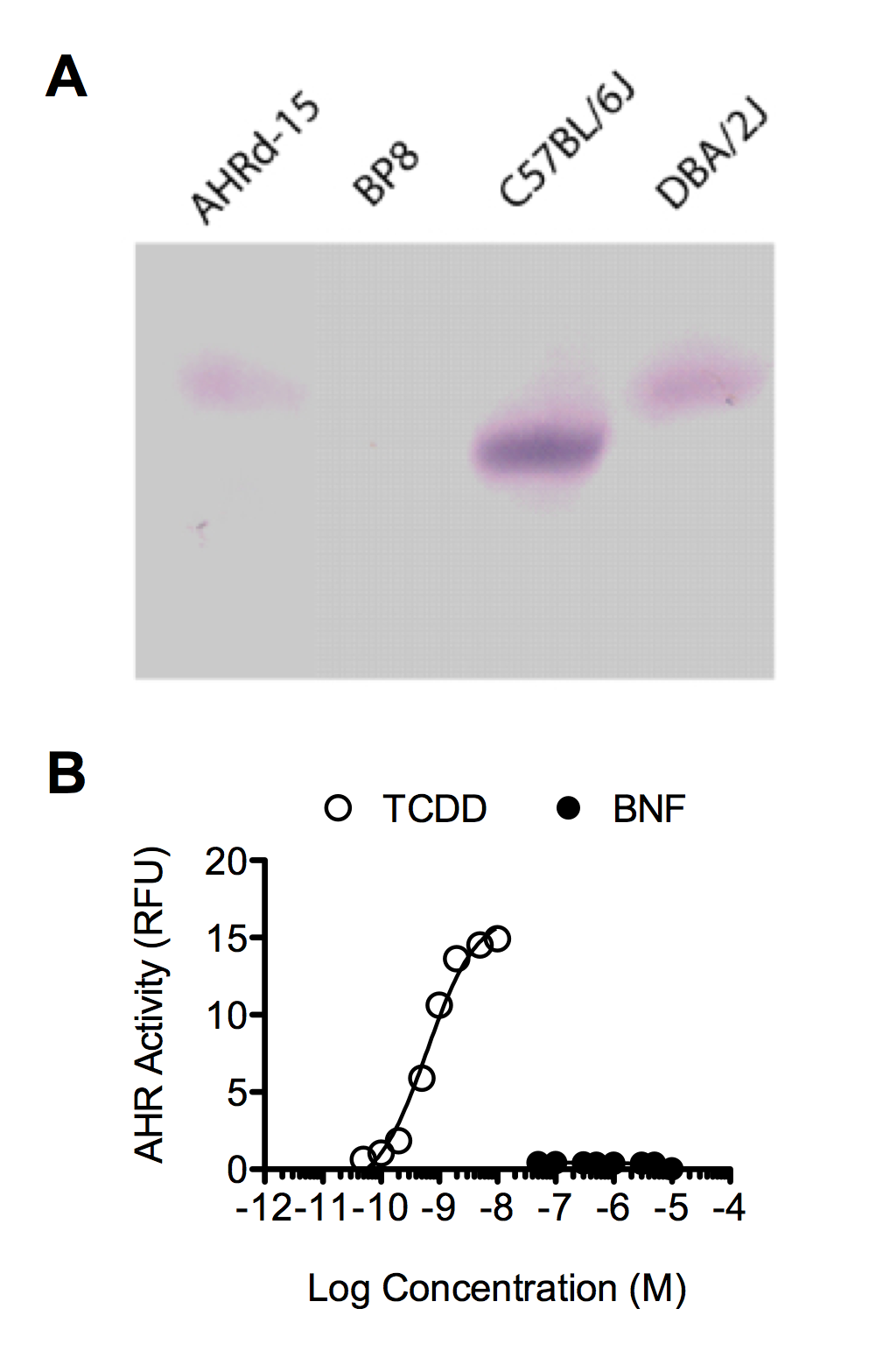

Supplement: Figure S1 — Characterization of AHRd-15 cell line. A. A Western blot was performed using the whole cell lysate of AHRd-15 cells. Lysate from the AHR null BP8 parental cell line, and the hepatic cytosolic fractions from C57BL/6J and DBA/2J mice were included as size controls. Proteins were resolved by electrophoresis on a 7.5% acrylamide gel, and then probed with the BEAR-3 anti-AHR antibody. B. AHRd-15 is responsive to TCDD, but not BNF. Dose-response curves were generated by treating AHRd-15 cells with nM doses of TCDD and µM doses of BNF for 36 hours. Activation of the AHRd was determined by quantifying EROD activity from whole cell lysate. (TIFF) [file pone.0044547.s001.tiff]

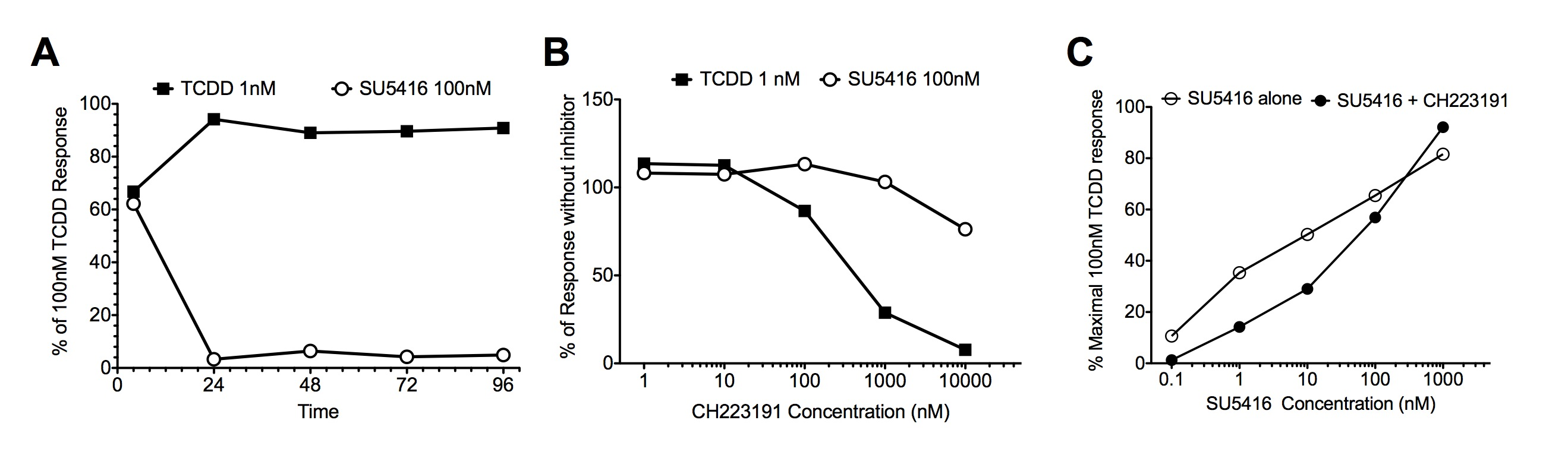

Supplement: Figure S2 — Duration of action of AHR activation. A. 0.6×10^6^ Cells from a mouse hepatoma cell line H1L6.1c3, stably carrying a dioxin-responsive element (DRE)-driven firefly luciferase reporter gene were seeded in each well of a six-well plate overnight and were then treated with SU5416 100 nM or TCDD 1 nM for 4 hours through 96 hours. DRE activity was assayed by a luminometer at the time points shown. Data is presented as a percent of 100 nM TCDD response at those time points. B–C. Characterization of antagonism of response of SU5416 by CH223191. B. SU5416 100 nM or TCDD 1 nM were tested with DRE-driven luciferase reporter cells with titrating doses of the antagonist for 4 hours as delineated in the figure. Data is presented as % response without inhibitor. C. SU5416 was titrated in culture with and without the antagonist (10 µM) for 4 hours. Results are presented as % maximal TCDD response at 100 nM. (TIFF) [file pone.0044547.s002.tiff]

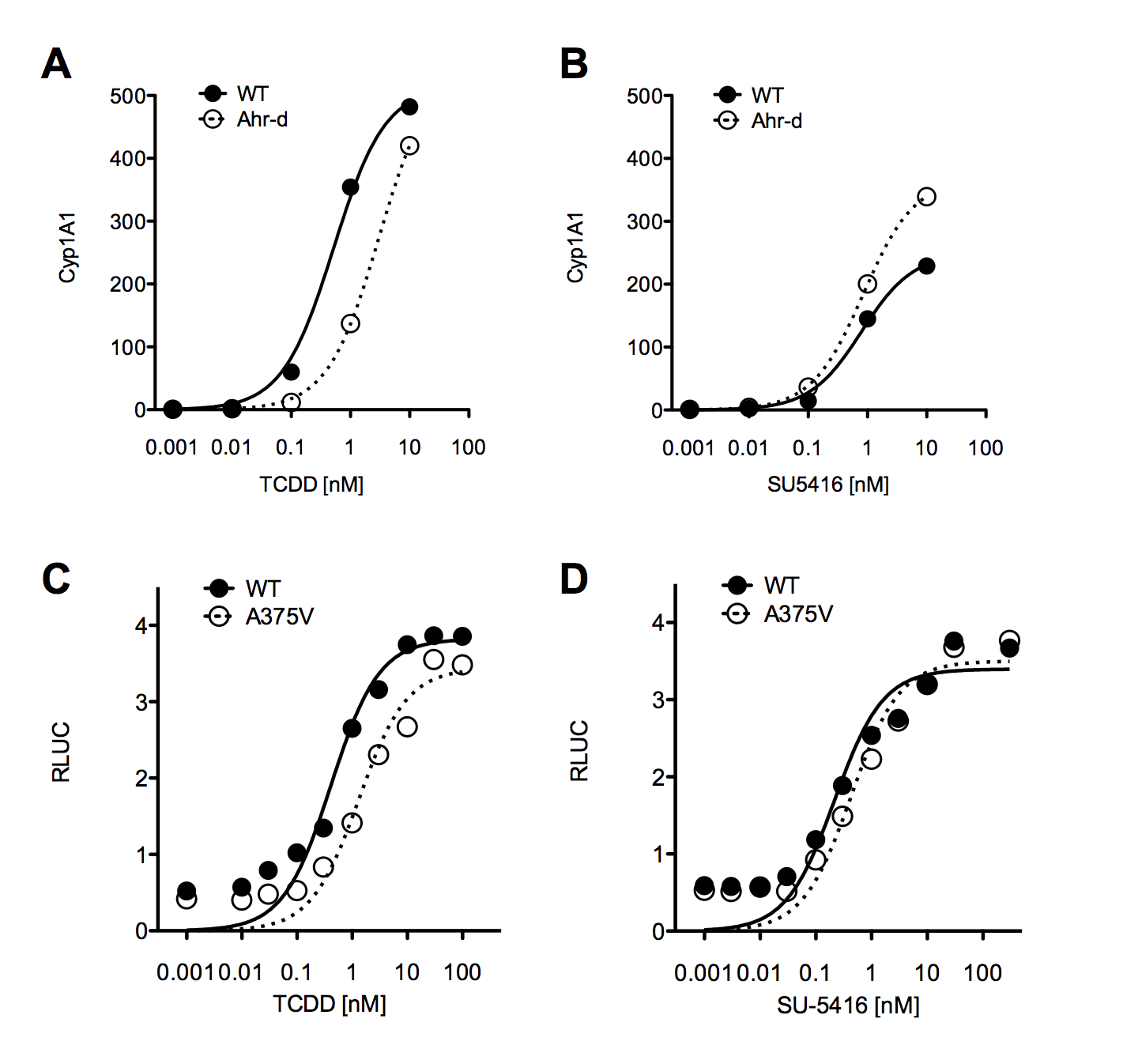

Supplement: Figure S3 — Similar to figure 4 , splenocytes from wild-type and AHRd mice analyzed by qPCR for CYP1A1. Spleens from these mice were harvested and suspended in culture media, and exposed to titrating doses of A) TCDD B) SU5416. After 4 hours they were analyzed by qPCR for CYP1A1 analysis. The curves represent fold change and show the similar potency of these ligands. Each graph is representative of 3 independent experiments. C–D. Cells transfected with AHR containing a valine point-mutation show similar ED50 to Cos-1 cells with AHRb isoform. Cos-1 cells were transfected with an AHR containing the same point mutation (valine for alanine) thought to be responsible for the low affinity of the AHRd isoform compared to AHRb, and compared to the wild-type AHR response. These cells also harbor a luciferase gene next to the DRE. C. Cos-1 cells were exposed to TCDD. D. Cos-1 cells were exposed to SU5416. The graphs represent true luciferase values. They are representative of 2 independent experiments. (TIFF) [file pone.0044547.s003.tiff]

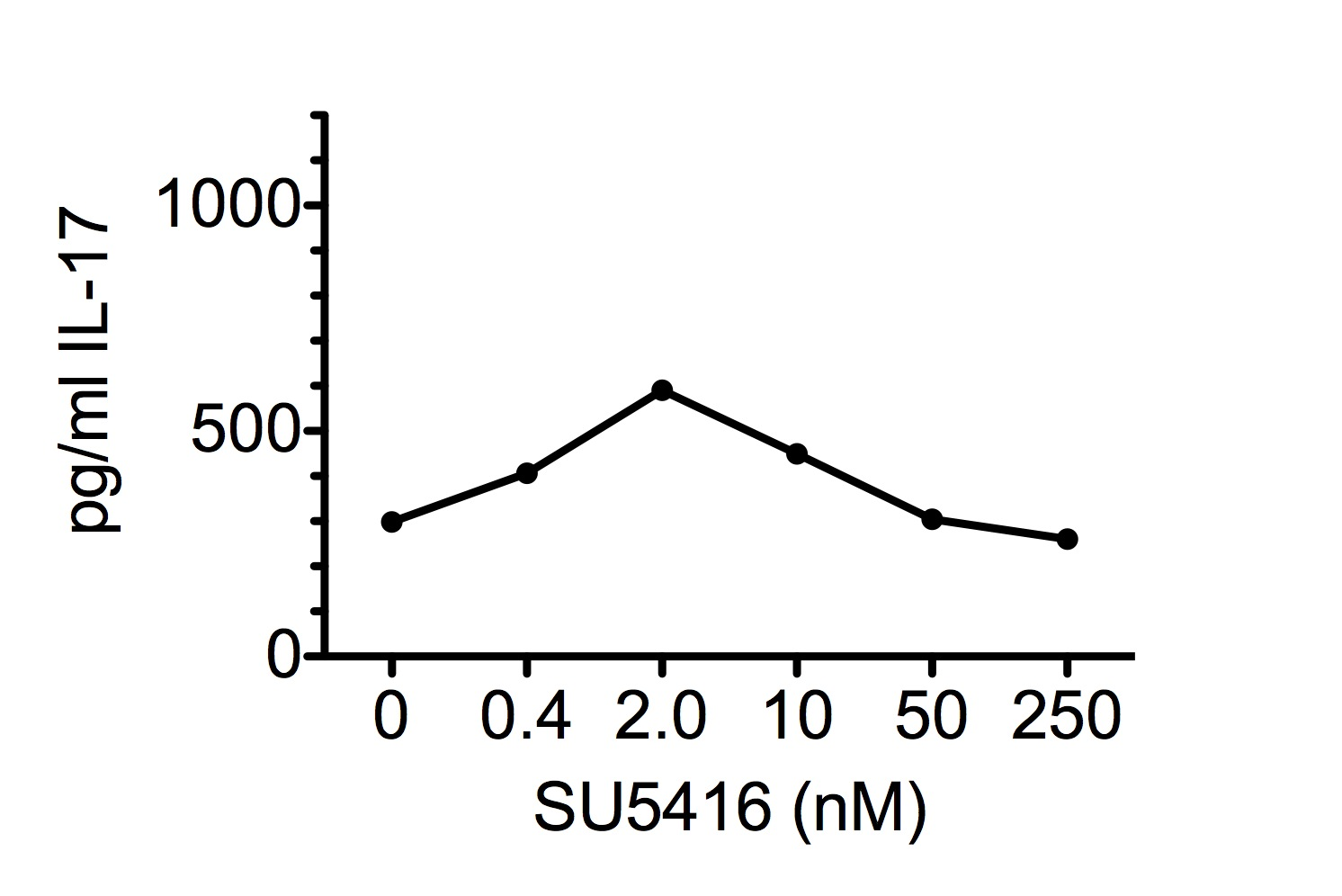

Supplement: Figure S4 — SU5416 causes a small amount of IL-17 secretion at low doses. Naïve T-cells were placed in Th17 conditions in culture (TGF-β 4 ng/ml, IL-6 20 ng/ml) and exposed to titrating doses of SU5416 as indicated. After 3 days of culture, supernatant was harvested and tested for IL-17 by ELISA. (TIFF) [file pone.0044547.s004.tiff]

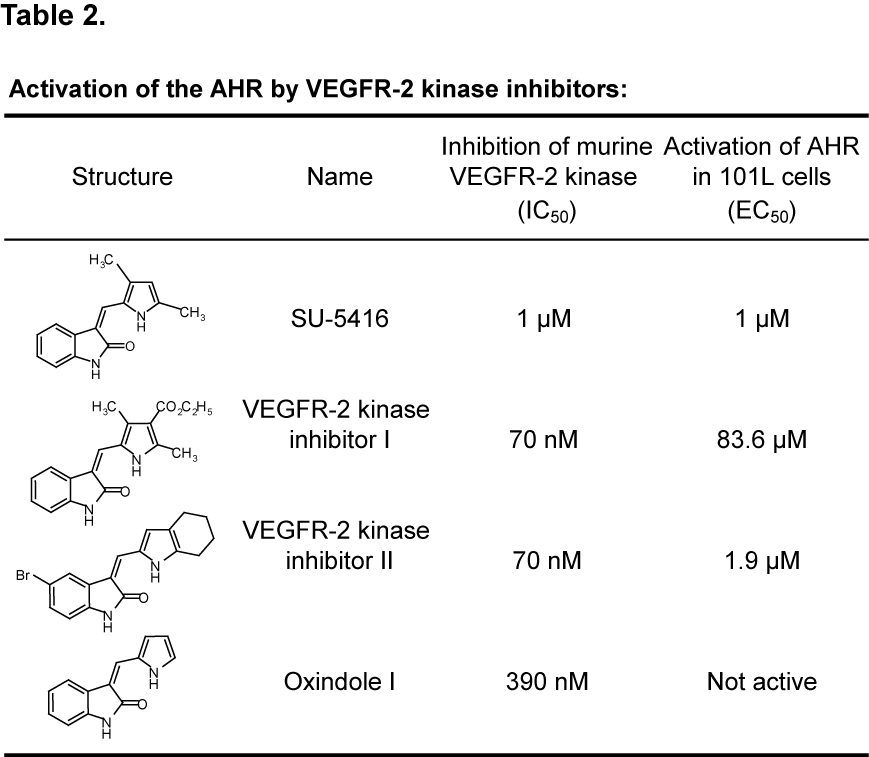

Supplement: Figure S5 — Activation of the AHR by VEGFR-2 kinase inhibitors. Different VEGFR-2 kinase inhibitors were tested for their ability to activate 101L cells in a luciferase assay, signifying AHR activity. Cells were incubated for 20 hours with mid-log concentrations of these compounds ranging from 0.01–30 µM. EC50 were calculated. These values were compared to reported IC50 of these compounds for inhibition of phosphorylation activity of VEGFR-2. (DOCX) [file pone.0044547.s005.docx]
